# Adaptation and resilience

**DOI:** 10.1186/s40985-016-0032-5

**Published:** 2016-10-10

**Authors:** Kristie L. Ebi

**Affiliations:** Departments of Global Health and of Environmental and Occupational Health Sciences, University of Washington, Seattle, Washington USA

**Keywords:** Health adaptation, Low- and middle-income countries, Climate change, Health outcomes, Implementation, Policy, Risk management

## Abstract

Lessons learned, opportunities, and barriers to scaling up health adaptation were identified from evaluation reports and other materials from three multinational health adaptation projects covering 14 low- and middle-income countries and from qualitative data collected through a focus group consultation and interviews with key informants purposively selected for their expertise and role in health adaptation. The national projects aimed to increase resilience to climate-sensitive health outcomes by focusing on incremental improvements in policies and programs to address climate variability and by beginning to establish enabling environments for further adaptation. At this early stage in implementing health adaptation, projects have made limited plans for scaling up specific health adaptation activities outside of normal ministry approaches. Scaling up is needed to prepare for the challenges ahead, including by improving integrated surveillance and other programs to manage the health risks of a changing climate.

## Background

The twenty-first century will be very different from the last. More countries than today will face challenges of food and water security. Environmental degradation is a growing problem worldwide, with adverse consequences for human health and well-being. Extreme weather and climate events cause injuries, illnesses, and deaths today, with their frequency, intensity, and in some cases duration expected to increase with climate change. These global environmental changes affect children: about 85 % of the health impacts of climate change are in children. The international negotiations under the United Nations Framework Convention on Climate Change (UNFCCC) recognize the importance of focusing on the risks to women and children, making sure the most vulnerable will be protected over coming decades as the climate continues to change.

## Main text

**Fig. 1:**
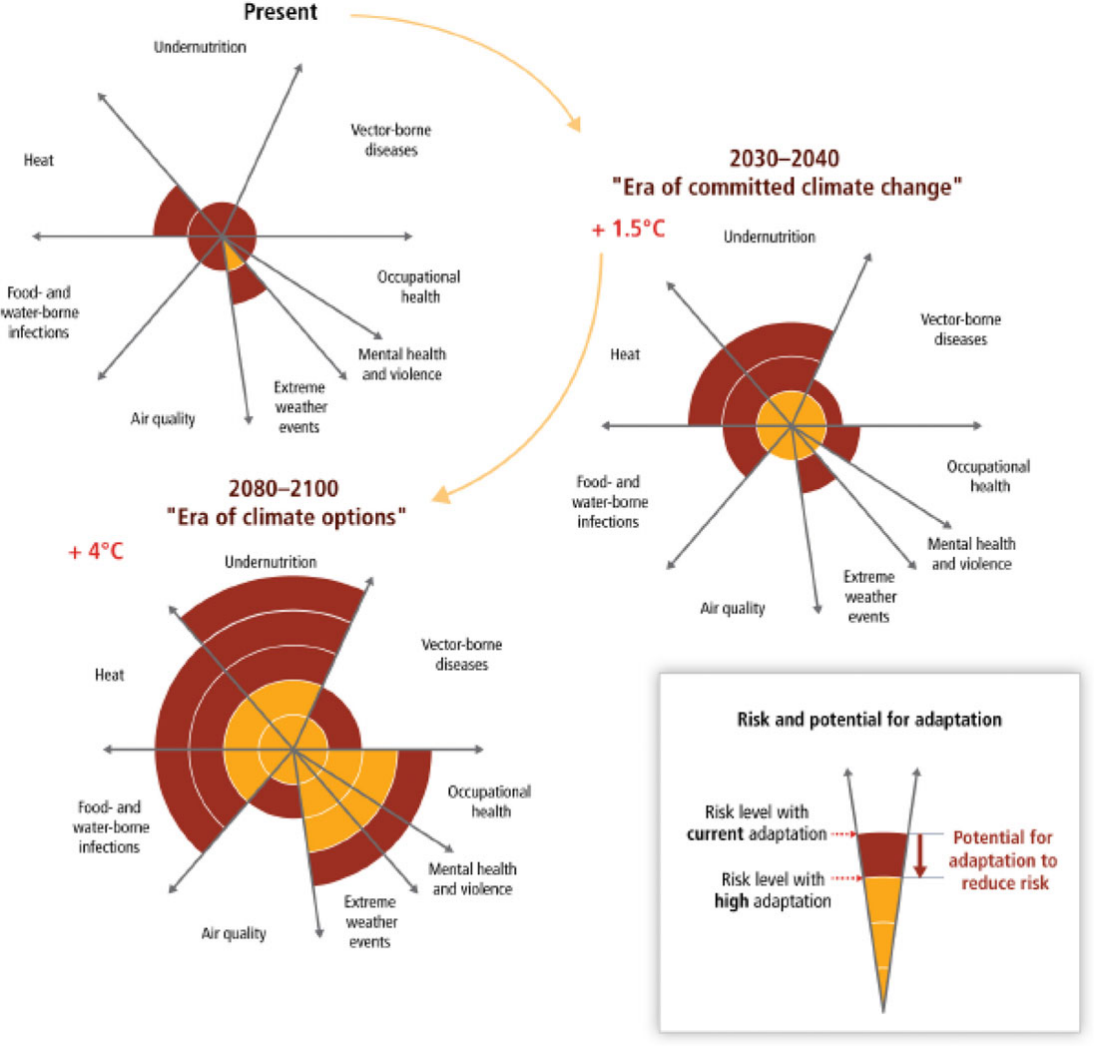
Conceptual presentation of the health impacts from climate change and the potential for impact reduction through adaptation. Source: Smith et al. [1]

Figure 1 from the Intergovernmental Panel on Climate Change (IPCC) 5th Assessment Report summarizes the potential for health adaptation to reduce risks, based on expert judgement and an assessment of the literature [1]. This slide summarizes several key messages relevant for adaptation and resilience. The figure in the upper left-hand corner, labelled the present, summarizes current risks and the potential for adaptation to better manage those risks. As shown in the legend, the red areas indicate the risk level with current adaptation (e.g. no additional efforts undertaken) and the gold areas indicate how much the risks could be reduced by effective, efficient, and proactive adaptation. The width of the wedges indicates the magnitude of the burden of major climate-sensitive health outcomes. Undernutrition and changes in the geographic range, seasonality, and intensity of transmission of vector-borne diseases are among the most important risks of climate variability and change today. As would be expected, risk levels are moderate today, with the potential to reduce risks for adverse health outcomes from extreme weather and climate events.

Looking across the periods 2030–2040 and 2080–2100, the figures show that risks are expected to increase dramatically over this century, with the opportunities for adaptation also increasing. However, even with proactive adaptation, there will be significant residual risks that will need to be managed, particularly for undernutrition, heat-related morbidity and mortality, and food- and water-borne infections. Ministries of health will need to quickly and efficiently implement proactive adaptation option and to prepare for large increases in the magnitude of some climate-sensitive health risks. Limited adaptation efforts over the next few years will increase the risks that will need to be managed by mid-century.

Positive news from a public health perspective is that the health concerns of a changing climate are known risks. Ministries of health already have programmes to address climate-sensitive health outcomes, such as malaria and diarrheal diseases; there are many tools, methods, and guidance documents for reducing and managing current and future health risks. Specific health adaptation projects are starting to be implemented, which will provide best practices and lessons learned to inform which interventions to scale up.

In addition to adaptation efforts within health systems, increasing resilience to climate change requires strong partnerships across sectors. The burdens of many health outcomes are not only a consequence of the effectiveness of policies and programmes within a ministry of health, such as for infectious diseases, but also are a consequence of policies and programmes in agriculture, water, and urban sectors. Collaborations across ministries are developing in many countries, with increasing numbers of effective examples. But much more progress is needed. Developing such collaborations will facilitate identifying and implementing innovative solutions to support transitions to more resilient and healthy societies.

The World Health Organization (WHO) published guidance on efforts needed to build climate-resilient health systems, summarizing the roles and responsibilities within a ministry of health [2]. Moving to climate-resilient health systems will require activities on many fronts, including enhancing leadership and governance to manage climate change; health workforce strengthening; conducting vulnerability, capacity, and adaptation assessments; developing integrated risk monitoring and early warning; conducting health and climate research; identifying and promoting climate resilient and sustainable technologies and infrastructure; improving management of the environmental determinants of health; developing climate-informed health programmes; increasing emergency preparedness and management; and increasing climate and health financing.

There is a significant opportunity for most health systems to increase their resilience to climate change. Current policies and programmes for managing climate-sensitive health outcomes were established without considering climate variability and change, resulting in a significant gap for achieving resilience. For example, in certain regions, malaria control programmes will likely need to consider how climate change could alter the geographic range of the vector and disease, seasonality, and the intensity of transmission, if they are to maintain their current level of effectiveness. Current and proposed surveillance and monitoring may need to be altered to include regions likely to be at risk in the coming years, which implies that projections of malaria risks are available at the appropriate scale. Consideration also will be needed of whether sufficient human and natural resources are available to make necessary changes to policies and programmes and whether partnerships with other ministries and organizations are needed to provide the required information (such as climate projections) to support informed decision-making.

WHO also published guidance on protecting health through adaptation planning to help these transitions to climate-sensitive policies and programmes [3]. The guidance on conducting the health component of a national adaptation plan promotes integration across sectors and highlights the importance of integration from local to national scales. This guidance is being used by ministries of health to develop national health adaptation plans that integrate with plans developed by other sectors and to ensure protection of population health in a changing climate.

Several international projects were funded over the last 5 to 7 years on health adaptation. Lessons learned are highlighted from a seven-country WHO/United Nations Development Programme (UNDP) health adaptation project funded by the Special Climate Change Fund under the UNFCCC [4]. Examples of successful outcomes from some of these projects are highlighted below.

Bhutan is a mountainous country experiencing dengue and malaria moving into highland areas. Each village has a volunteer healthcare worker who undergoes training every year. Over the course of the WHO/UNDP project, healthcare workers from pilot communities were trained on the risks of climate change and on solutions to better manage changing burdens of disease. In a discussion several years ago, these workers, primarily farmers, talked about their lifetime experiences, what they had observed, and how it related to climate change. One farmer said when he was a child, he could always tell when it was time for the fall festival because there was snow on the mountains. There no longer is snow on the mountains for the fall festival. He does not remember seeing mosquitos when he was a child. Whether or not there were mosquitos, mosquitos were not an important consideration as he was growing up. In April 2013, his community was sleeping under mosquito bed nets and the hospital said the closest known case of malaria was just 10 km away. So, in his lifetime, he has gone from not seeing diseases like dengue and malaria to being at risk of them.

The WHO/UNDP project in Bhutan was very impressive; it facilitated integration of data and information from the ministry of health with the meteorological services to develop early-warning systems. Through the project, collaborations were developed across the government to address challenges associated with a changing climate, and partnerships strengthened with departments working directly with communities, to ensure information was communicated to those most at risk.

Another country in the project was Jordan, which is one of the ten most water-stressed countries in the world, with significant challenges of water security. Starting several years ago, treated wastewater was used for agricultural irrigation. The neighbouring communities began experiencing higher than normal rates of diarrhoeal disease. The project facilitated coordination across the many departments and ministries with differing roles and responsibilities for water safety and security in Jordan. Achieving coordination across multiple mandates was challenging. In addition, the project supported underlying research that recently showed it was unlikely the treated wastewater was causing the increase in diarrhoeal disease; water handling and other issues were more important. The project aimed to ensure that Jordanians would have access to safe water while infrastructure is transitioning to a world where using treated wastewater will be commonplace.

Barbados, a third country in the WHO/UNDP health adaptation project, among other activities, trained schoolchildren about climate change and health. The children developed posters showing what could be done in Barbados to reduce their carbon footprint.

An example of reducing greenhouse gas emission comes from Thailand. A medium-sized, 250-bed hospital outside of Bangkok (17th Somdejphrasangkharaj Hospital) implemented a CLEAN (Communication, Leader, Effectiveness, Activity, Networking) and GREEN (Garbage, Restroom, Energy, Environment, Nutrition) programme, with activities for each to promote resilience and sustainability. The hospital tracks its daily greenhouse gas emissions and has an extensive reuse program. The hospital staff designed and implemented a number of innovative activities. For example, a path was built around a wastewater treatment pond so that patients and staff could exercise. In addition, three bicycles were installed: riding the first aerates the pond, needed when treating wastewater; the second pumps pond water into a tub; and the third waters the lawn using a sprinkler system attached to the tub. In 2011 alone, the hospital reduced their greenhouse emissions by an impressive nearly 14 % with a low-cost set of activities. The hospital has won many well-deserved awards and shows what can be done locally with leadership and ingenuity. There are increasing examples of regional and local activities promoting more resilient societies using what they have at hand.

## Conclusions

Climate change presents many risks to population health that, when addressed, could increase societal resilience and sustainability. In addition to adaptation and mitigation efforts, additional human and financial resources will be needed to prepare for and prevent the burdens of climate-sensitive health outcomes from increasing in future decades. Irrespective of resource constraints, low- and middle-income countries need to prepare for climate change through better understanding of potential risks, strengthening health systems, ensuring adequate policies and legislation, facilitating institutional support, and public education and awareness programs, including disaster preparedness measures.

### Questions

#### Speaker from the floor

Madeleine Thomson from Columbia University. Thank you very much for the panel, I thought it was great and I was really interested to see a focus on the variability of the climate as well as a particular focus on Africa. I still think there is a gap that we have in the development of climate change and health where the benefits of a mitigation strategy for health are very clear in developed countries, in rapidly urbanizing environments, etc. and particularly in Asia, but less so, the discussion I think has been less developed for Africa and particularly the challenge we have around managing climate variability, which is now integrating with climate change. And I take for example the current El Niño, the biggest drought that we have in Ethiopia, which is really, meteorologically, the largest drought, bigger than the drought in 1984, and many of you in this room will remember the impact of that drought. We will see over the coming year, really, the capacity of the Ethiopian government to manage that drought and the donor response. But if we don’t see that as part of climate change response, I think that we are really going to miss out, and particularly in the African context so I would really like to emphasize keeping an eye on that and also building that response in a more integrated way into the climate change discussion.

Thank you.

#### Speaker from the floor

Alex MacMillan from the New Zealand Climate and Health Council. It is great to see the development of systems thinking in planetary health and, as a systems-modelling environmental-health person myself, it is really reassuring to see. I wonder whether the ongoing calls for systems thinking and planetary health over time are about a complete lack of capacity among public health researchers, so my question really is about how we move public health research into systems thinking and capacity build in that way, and what’s happening there.

#### Kristie Ebi

Thank you. Those are very good comments illustrating the challenges when a speaker has ten minutes to cover a broad field. Systems thinking is critical to addressing the health risks of climate and other global environmental changes. It’s very positive that some funders are beginning to move this approach forward; further efforts are needed.

The comment on soils: it’s not so much a lack of recognition in the health sector of the important of soils. A major challenge is the disconnect between agriculture and health systems. Agricultural models projecting the risks to food security typically focus on crop yields. There is a gap between these projections and health systems modelling of the risks of undernutrition with climate change. This goes back to the point about systems thinking; the importance of integrating across sectors to better understand risks and responses.

There have been efforts over the past twenty-some years to explicitly include climate variability in the UNFCCC and the IPCC. What I was hoping Dr. Thomson was going to say was that there are many researchers in this room; with the current El Niño, now is a perfect time for people to set up experiments to quantify the health risks of such events. If you have got long-term data sets, please think about how you can take advantage of this natural experiment, and come back next year to tell us what you found.

## Abbreviations

IPCC: Intergovernmental Panel on Climate Change
UNDP: United Nations Development Programme
UNFCCC: United Nations Framework Convention on Climate Change
WHO: World Health Organization

## References

[1] Smith KR, Woodward A, Campbell-Lendrum D, Chadee DD, Honda Y, Liu Q, et al. Human health: impacts, adaptation and co-benefits. In: Field CB, Barros VR, Dokken DJ, Mach KJ, Mastrandrea MD, Bilir TE, et al., editors. Climate change 2014: impacts, adaptation, and vulnerability. Part A: Global and sectoral aspects. Contribution of Working Group II to the Fifth Assessment Report of the Intergovernmental Panel on Climate Change. Cambridge and New York: Cambridge University Press; 2014.

[2] World Health Organization. Operational framework for building climate resilient health systems. 2015. http://www.who.int/globalchange/publications/building-climate-resilient-health-systems/en/. Accessed 6 Oct 2016.

[3] World Health Organization. WHO guidance to protect health from climate change through health adaptation planning. 2013. http://www.climateandhealthalliance.org/asset/download/78/1529%20Health%20National%20Adaptation%20Process%20121113.pdf. Accessed 6 Oct 2016.

[4] World Health Organization (2015). Lessons learned on health adaptation to climate variability and change: experiences across low- and middle-income countries.

